# Ovary Transcriptome Profiling via Artificial Intelligence Reveals a Transcriptomic Fingerprint Predicting Egg Quality in Striped Bass, *Morone saxatilis*

**DOI:** 10.1371/journal.pone.0096818

**Published:** 2014-05-12

**Authors:** Robert W. Chapman, Benjamin J. Reading, Craig V. Sullivan

**Affiliations:** 1 Marine Resources Division, South Carolina Department of Natural Resources, Charleston, South Carolina, United States of America; 2 Marine Genomics Core Facility, Hollings Marine Laboratory, Charleston, South Carolina, United States of America; 3 Department of Applied Ecology, North Carolina State University, Raleigh, North Carolina, United States of America; 4 Department of Biology, North Carolina State University, Raleigh, North Carolina, United States of America; Glasgow Caledonian University, United Kingdom

## Abstract

Inherited gene transcripts deposited in oocytes direct early embryonic development in all vertebrates, but transcript profiles indicative of embryo developmental competence have not previously been identified. We employed artificial intelligence to model profiles of maternal ovary gene expression and their relationship to egg quality, evaluated as production of viable mid-blastula stage embryos, in the striped bass (*Morone saxatilis*), a farmed species with serious egg quality problems. In models developed using artificial neural networks (ANNs) and supervised machine learning, collective changes in the expression of a limited suite of genes (233) representing <2% of the queried ovary transcriptome explained >90% of the eventual variance in embryo survival. Egg quality related to minor changes in gene expression (<0.2-fold), with most individual transcripts making a small contribution (<1%) to the overall prediction of egg quality. These findings indicate that the predictive power of the transcriptome as regards egg quality resides not in levels of individual genes, but rather in the collective, coordinated expression of a suite of transcripts constituting a transcriptomic “fingerprint”. Correlation analyses of the corresponding candidate genes indicated that dysfunction of the ubiquitin-26S proteasome, COP9 signalosome, and subsequent control of the cell cycle engenders embryonic developmental incompetence. The affected gene networks are centrally involved in regulation of early development in all vertebrates, including humans. By assessing collective levels of the relevant ovarian transcripts via ANNs we were able, for the first time in any vertebrate, to accurately predict the subsequent embryo developmental potential of eggs from individual females. Our results show that the transcriptomic fingerprint evidencing developmental dysfunction is highly predictive of, and therefore likely to regulate, egg quality, a biologically complex trait crucial to reproductive fitness.

## Introduction

Reproductive fitness is a key issue in evolutionary biology and one of the limiting components of reproduction is the formation of viable gametes. In wild and domestic animals, egg quality is affected by many factors and can be highly variable, with production of inviable eggs being common in many species, including humans. Thus, poor egg quality, defined as the inability of developmentally incompetent eggs to produce viable embryos, is a serious problem faced in agriculture and human reproductive medicine that has persisted despite decades of attention.

The earliest stages of vertebrate embryo development are characterized by rapid, synchronous cell divisions subdividing the zygote into a large population of blastomeres termed a ‘blastula’. During this time, the developmental competency and viability of the nascent embryo is governed by crucial maternal RNAs that are deposited in growing oocytes to direct early embryogenesis. Zygotic transcription is initiated later, with the timing of the maternal-to-zygotic transition of transcription (MZT) dependent on the taxa [1]. In fish and other less derived vertebrates, the MZT involves a ‘midblastula transition’ (MBT) to longer, asynchronous, cell cycles that is accompanied by the activation of embryonic transcription [2]–[4].

The total dependency of early vertebrate embryogenesis on maternal mRNAs has prompted investigations of the role(s) of the inherited transcriptome in determination of egg quality with regard to embryo developmental potential. Assessments of human oocyte transcriptomes and their relation to egg quality are at an early stage of understanding and there is still not enough data on gene expression patterns in oocytes and blastomeres to distinguish high and low quality embryos, although considerable progress along these lines is being made in both human and mammalian animal models [5].

Amongst lower vertebrates, the transcriptomics of egg quality has been most extensively studied in fishes [6]–[8]. This focus arises from the fact that poor egg quality is a major limiting factor for development of global finfish aquaculture [9]–[11], and it is also due to the emergence of the zebrafish (*Danio rerio*) as a model for reproductive transcriptomics [12]–[14].

The persistence of some maternal mRNAs, and the transition from maternal to zygotic transcriptome, extend well past MBT in zebrafish, with specific functional subsets of transcripts showing fast, intermediate or slow degradation during early development [13], [15]. Although there are many exceptions, most maternal gene transcripts persisting until MBT are involved in the cell cycle and its regulation, mitosis, and nucleoskeletal changes during cell division (i.e. in predominant cytological events occurring during this time). Likewise, the oocyte-specific module of transcripts present during human and mouse early pre-implantation development up until MZT is mainly involved with these same cytological processes [16].

Transcriptomic profiling of the developmental potential of mammalian oocytes/embryos has heretofore been limited by the scant material available to assess RNA levels, and most studies have focused on transcripts present in cumulus or granulosa cells isolated along with oocytes. Therefore, information on what constitutes the transcriptome of a ‘high quality’ oocyte or embryo is presently lacking. The advent of ‘single-cell transcriptomics’ applied to individual oocytes [16], blastomeres [17], or polar bodies [18] may eventually remedy this deficiency.

Molecular studies of fishes, especially rainbow trout (*Onchorynchus mykiss*), have identified scores of maternal mRNAs that are correlated with egg quality or that significantly differ in expression between eggs of varying quality [8], [19], and levels of several transcripts were shown to be responsive to maternal treatments that alter egg quality, such as hormone-induced or photoperiod-advanced spawning, or postovulatory ageing of the eggs [20]–[22]. As examples, these transcripts encode regulators or participants in cell cycling (cyclins, nucleoplasmin), proliferation, growth and apoptosis (insulin-like growth factors and their receptor, prohibitin) and cytoskelton (tubulin beta, keratins 8 and 18). However, a comprehensive picture of the transcriptome of a developmentally competent fish oocyte or egg remains to be developed [23].

The present study was undertaken to identify critical component(s) of the ovary transcriptome that underpin egg quality in farmed striped bass (*Morone saxatilis*), a species with significant egg quality problems [24]. The study focused on females whose oocytes and eggs all appear to develop normally but whose spawns differ widely in rates of successful embryo development. We employed artificial neural networks and supervised machine learning [25]–[26] to discover the minimum constellation of transcripts whose collective expression best predicts egg quality, followed by modulated modularity clustering to visualize the component modules of co-expressed genes [27], and pathway analyses to identify gene networks within modules. This novel approach allowed us to identify, for the first time in any vertebrate, a transcriptomic “fingerprint” evidencing specific molecular dysfunctions that is highly predictive of, and therefore likely to determine, egg quality.

## Materials and Methods

### Ethics Statement

This study was carried out in strict accordance with the recommendations in the Guide for the Care and Use of Laboratory Animals of the National Institutes of Health. The animal use protocol was approved by the Institutional Animal Care and Use Committee of North Carolina State University (Protocol number 10-042-A). No animals were sacrificed in the course of this research, all invasive procedures (e.g. ovarian biopsy) were performed under tricaine methanesulfonate (MS222) anesthesia, and all efforts were made to minimize suffering.

### Experimental Animals, Tissue Sampling, and Spawning Procedures

Four-year-old adult female striped bass (*Morone saxatilis*) from the founder stocks of the National Program for Genetic Improvement and Selective Breeding for the Hybrid Striped Bass Industry (National Breeding Program) held at the NCSU Pamlico Aquaculture Field Laboratory (Aurora, NC) were subjected to ovarian biopsy antecedent to the spawning season in April-May, 2010, and were tagged with passive integrating transponders (PIT; Destron, IDI). Biopsy samples were aspirated into a plastic cannula inserted through the urogenital pore [28] and expelled into cryovials containing RNA*later* (Ambion), held at 4°C overnight, and then decanted and stored frozen at −20°C until being extracted of RNA for microarray. The biopsies were aspirated from ovarian lamellae containing mostly oocytes in intact follicles and they consisted of ≥55 mg of tissue containing ≥60 oocytes, which were estimated to make up over 95% of the tissue mass. An aliquot of the biopsy samples was examined under a stereomicroscope and females whose most mature oocytes exceeded 1000 µm diameter and appeared to have a uniform size-frequency distribution with no signs of onset of preovulatory atresia were then moved into the hatchery, warmed to and held at spawning temperature (18°C) for 7–10 days and then implanted with 95% cholesterol pellets containing a synthetic analog of gonadotropin-releasing hormone (GnRHa) to induce final ovary maturation and ovulation [29]. Induced spawning and assessments of egg quality followed routine striped bass hatchery procedures [28]. Only females that progressed normally to spawning according to standard culture procedures (i.e., were maturationally competent) were used for the experiment. Briefly, eggs were stripped from ovulated females, fertilized with semen pooled from three males of the same year class, water hardened, and incubated in standard MacDonald hatching jars set up so that progeny from each female hatched into individual aquaria. For each female, total body length and weight, fecundity, the percent of eggs bearing well-formed embryos at 4 hours (h) and 24 h post-fertilization, the percentage of 24 h embryos hatching, and the percentage of hatched larvae surviving for 5 days (d) were recorded. Fin clips were taken from each female and stored in 70% ethanol for microsatellite genotyping.

### Measures of Striped Bass Spawn Quality

Female striped bass were sorted into groups (N = 8 each) producing ‘high quality’ or ‘low quality’ eggs (spawns) based upon the percentage of eggs bearing viable 4 h embryos. Spawns with >50% of eggs producing 4 h embryos were considered to be of high quality and spawns with <30% of eggs producing 4 h embryos were considered to be of low quality. Individual embryos were considered viable if they exhibited normal cell cleavage, symmetry, and form. Differences between female fish in the high and low egg quality groups in body length and weight, fecundity, maximum oocyte diameter at initial biopsy, percent of viable embryos at 4 h and 24 h post-fertilization, hatching rate of 24 h embryos, and survival of hatched larvae to 5 days post hatch (dph) were evaluated using a Student's t-test, with proportional data being arcsin-square root transformed prior to statistical analysis.

### Striped Bass Genotyping and Pedigree Analyses

Genotyping and pedigree analyses were performed in the Fisheries and Aquaculture Molecular Genetics Laboratory at the Virginia Institute of Marine Science (Gloucester Point, VA). Whole genomic DNA was extracted from tissue samples (fin clips) taken from striped bass females using a DNAeasy Kit (Qiagen) and used to amplify 48 microsatellite loci (**Table S1**) via PCR [30]–[31]. These loci were from a set of 289 loci recently used to develop a medium density genetic linkage map for striped bass [32] and were selected to maximize exhibited allelic variability and genome coverage. Alleles were scored using GeneMarker software v. 1.75 (SoftGenetics LLC) and constructed genotypes for the fish were entered into a spreadsheet, formatted using MAKEPED for the LINKAGE program [33] and checked for inconsistencies with Mendelian inheritance using PedCheck [34]. Resulting data were used to verify the pedigree of females to the level of family using the software program COLONY [35]. A digital matrix showing the scoring of all alleles (0 = absent, 1 = one copy, 2 = 2 copies) for each individual served as input for analysis of the microarray data via artificial neural networks (ANNs) and machine learning tools (see below).

### Striped Bass Ovary UniClone Microarray Development and Procedures

Using 454 Pyrosequencing (Roche), we developed a transcriptome database for striped bass comprised of 230,151 expressed sequence tags (ESTs) derived from ovary cDNAs that included all maturational stages and ovulated eggs (National Center for Biotechnology Information (NCBI) Accession: SRX007394) [36]. The assembled and annotated contiguous EST sequences (11,208 contigs) are available at the U.S. National Animal Genome Project website (http://www.animalgenome.org) and were used to design an Agilent Technologies 8×15,000 feature, 60-mer oligo microarray (eArray Group, Striper Group; Design ID, 029034), which included 11,145 UniGene probes from the ovary transcriptome, plus 3,854 probes printed as duplicates or selected from *Morone* cDNAs available at the NCBI. Microarray procedures followed those of Chapman and associates [25]–[26]. Briefly, total ovary RNA (39.2+1.8 µg; mean+SEM) was isolated from biopsy samples stabilized in RNA*later*, assessed for concentration and quality using the 2100 Bioanalyzer (Agilent) and NanoDrop ND-1000 Spectrophotometer (NanoDrop Technologies), and used to produce a Cy3-labeled complimentary RNA (Cy3-cRNA) probe (6.1+0.2 µg; mean+SEM) to be hybridized to microarray slides, which were washed, dried and scanned with an Agilent Array Scanner (Model #G2505B) using Agilent Scan Control software. The scanned images were extracted with Feature Extraction v 10.7.3.1, Protocol GE1_107_Sep09.

Microarray gene expression data are deposited in the Gene Expression Omnibus (GEO, www.ncbi.nlm.nih.gov/geo/) at the National Center for Biotechnology Information (Series GSE42804, Platform GPL16363) according to Microarray Gene Expression Data Society Standard on Minimum Information about a Microarray Experiment.

### Microarray Data Analyses Using Artificial Intelligence

Analysis of microarray data utilized artificial neural networks (ANNs) and support vector machines (SVM). Variance-stabilized normalization (VSN) of the raw intensity data was achieved using Bioconductor (http://www.bioconductor.org/). Dimensionality of the VSN data was reduced by eliminating genes (inputs) unlikely to be associated with the output using a t-test to compare levels of gene expression between the binary categories of ‘high’ versus ‘low’ egg quality spawns or a linear regression equivalent in the case of continuous variables (e.g., percent of eggs bearing viable 4 h embryos). In order to select a tractable number of genes to initially focus on in the subsequent analyses by ANNs [25]–[26], we arbitrarily used *P*≤0.10 as a convenient cutoff, yielding about 10% of transcripts (N = 1469), which were arranged in rank order by *P*-value. Twenty (20) ANN models of the relationship between gene transcript levels and percentage of eggs bearing viable 4 h or 24 h embryos were developed using expression data from the selected 1469 probes, each model being trained on data from a random sample of 12 of the 16 fish (75% of the observed data). Following model training, gene expression data from the remaining 4 fish (25% of observed data), to which the models were naïve, was respectively entered for cross-validation (CV) to test model robustness. We then selected the top 250 and top 100 probes with the highest sensitivity values to similarly train and test two additional sets of 20 ANN models. Differences among mean model R^2^ values and among mean CV R^2^ values generated using the 1469, 250 or 100 probe data sets were detected by one-way analysis of variance of the arcsin-square root transformed R^2^ values and identified using Duncan's new multiple range test. The mean CV R^2^ values were compared statistically in the same manner. Additionally, we calculated the sensitivities of model outputs to changes in expression level of each individual gene. This is analogous to calculating the coefficient of variation in linear regression in order to assess the relative importance of independent variables.

### Graphic Representation of the Microarray Data

Hierarchical clustering heat maps for the 233 genes (top 250 probes - 17 duplicates) identified by ANN modeling were generated using the clustergram function available in MATLAB. Modulated modularity clustering (MMC) was used to profile ovary gene expression and to assign genes to transcriptional units subsequent to the ANN [27], [37]. Genes (n = 233) significantly correlated to spawn quality were reserved and the correlates among transcripts attributed to spawn quality were removed. To accomplish this, we reversed the ANN modeling (mapping fertilization success to individual gene expression levels) for the 233 most informative UniGenes and the residuals were extracted and submitted to MMC (http://mmc.gnets.ncsu.edu/). Relevance association gene networks also were generated from the transcriptional modules using a locally written MATLAB program to select those with (|r|≥0.6), which were then fed to Cytoscape (www.cytoscape.org) to generate the relevance networks.

### Annotation of 233 UniGenes Correlated to Spawn Quality

Sequences were subjected to BLAST (blastx) [38] of the NCBI database and annotated according to the Gene Ontology Consortium [39] using Blast2GO 2048M version 12.2.0 [40]–[41]. Parameters for blastx were: Expect value 1.0E-3 and HSP Length Cutoff 33. Parameters for the GO annotations were: E-value-hit-filter 1.0E-6, Annotation Cutoff 55, GO Weight 5, and HSP-Hit Coverage Cutoff 0. These GO assignments and the individual gene information provided by NCBI UniGene, Human Genome Organization Gene Nomenclature Committee (HGNC), and GeneCards [42] were utilized to assess potential gene functions and networks and their involvement in physiological processes relevant to egg quality.

## Results

**Figure 1:**
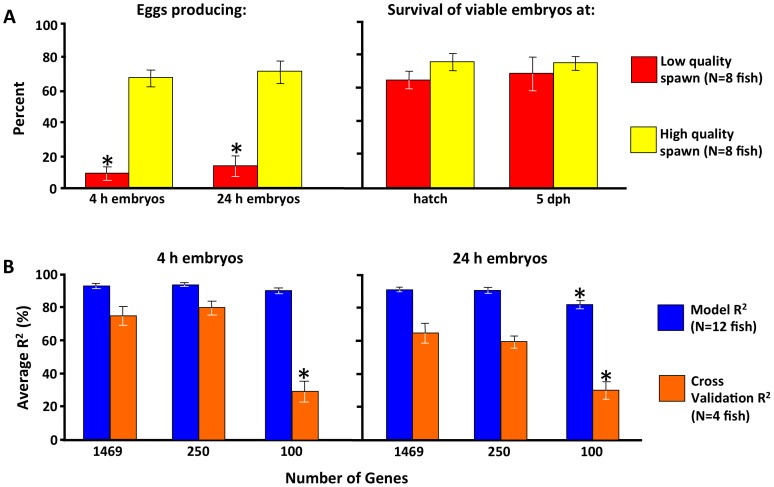
Results of analyses of spawn quality and its relationship to ovarian gene transcript profiles. (A) Mean percent of eggs producing viable 4 hour (h) and 24 h embryos, percent of viable 24 h embryos to hatch, and percent of hatched embryos surviving to 5 days post-hatch (dph). Vertical brackets indicate S.E.M. and asterisks indicate a significant difference (*P*<0.001) from values for high quality spawns. (B) Results of artificial neural network (ANN) analyses of the microarray data. Bars indicate mean R^2^ values for 20 ANN models, vertical brackets indicate S.E.M., and asterisks indicate a significant difference from the value obtained using the top 1469 genes (*P*<0.05).

There were no significant differences between females spawning high and low quality eggs in body length (mean +S.E.M.; 583.6 cm+7.1), weight (3.35 kg+0.11), fecundity (192,602 eggs/kg+13.497), or oocyte diameter (1079 µm+14) when initially biopsied (*P*>0.435), or in microscopic appearance of the biopsy samples or ovulated eggs (data not shown). All significant losses of embryos occurred within the first 4 h after egg fertilization (**Fig. 1A**). The percent of eggs from high quality spawns that produced 4 h embryos (67.3+5.1%) and 24 h embryos (70.1+6.8%) were significantly (*P*<0.001) and substantially greater than corresponding values for low egg quality spawns (9.0+4.1% and 13.4+6.2%, respectively). There were no differences between low and high egg quality spawns in hatching rate of 24 h embryos (66.5+5.7%) or larval survivorship of hatched embryos to 5 dph (67.9+6.6%) (**Fig. 1A**).

The mRNA extracted from ovary biopsies taken before the spawning season was subjected to microarray and results for the most informative 1469, 250 and 100 probe datasets were analyzed using ANNs to model the relation of ovary transcriptome to egg quality. These models on average explained >90% of the variation in 4 h and 24 h embryo survival (mean R^2^ range 0.902–0.930), with the exception of the 100 probe dataset model for 24 h embryo survival, for which 82.7% of the variation was explained (**Fig. 1B**). The mean CV R^2^ values for these models, a measure of the robustness of the ANN model based on its ability to predict egg quality, indicated that nearly 80% of variation in 4 h embryo survival (mean CV R^2^ = 0.777) and nearly 60% of variation in 24 h embryo survival (mean CV R^2^ = 0.596) could be predicted from gene expression measured using the top 250 probe dataset. Expanding the model training suite to include 1469 gene probes whose expression was most sensitive to egg quality did not improve mean model or CV R^2^ values and, when model training was constrained to the top 100 probes with the lowest *P* values, the predictive ability of the models decreased as evidenced by the significantly lower mean R^2^ for the respective CVs (**Fig. 1B**).

While collective changes in ovarian expression of a limited suite of probes (250), representing <2% of the queried ovary transcriptome, explained >90% of the variation in embryo survival, the differences in egg quality related to minor changes (<0.2-fold) in expression of any individual gene (**Table S2**). Additionally, the calculated sensitivities of model outputs to changes in the expression level of each gene showed that most transcripts made minor individual contributions to overall prediction of egg quality (<1% of the variation). The pedigree of female striped bass, evaluated by genotype at 48 microsatellite loci, did not strongly influence egg quality, explaining <1% of the variation.

**Figure 2:**
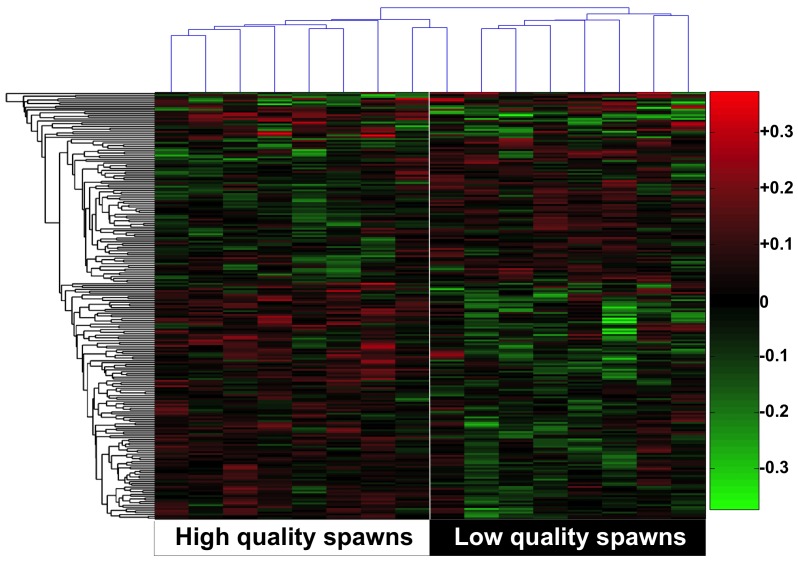
Two-dimensional hierarchical clustering heat map of the microarray data showing transcript expression profiles for genes (n = 233) expressed in ovary biopsies of female striped bass that produced low and high quality spawns. Levels of gene expression are indicated on the color scale to the right with numbers indicating the fold difference from the grand mean for all fish (shown in black and indicated by the zero value), red indicating increased transcript expression, and green indicating decreased transcript expression. Fish producing high or low quality spawns are grouped as indicated at the bottom of the figure. The clustering of individual genes with respect to their similarity in changes of expression between individual fish is represented by the dendrogram to the left. The dendrogram at the top shows similarities in gene expression patterns between individual fish regardless of group.

When seventeen (17) duplicates were removed from the informative 250 probe dataset and expression of the remaining 233 UniGenes was visualized on a hierarchical clustering heat map (**Fig. 2**), clear differences between ovary transcriptomes of females producing high versus low egg quality spawns were revealed. In general, ovary transcripts up-regulated in females producing low egg quality spawns were down-regulated in females producing high egg quality spawns (n = 145), and *vice versa* (n = 88) (**Table S2**). The heat maps also revealed some variation in expression of discrete genes between individual fish within the two egg quality groups. Additionally, the majority (62.2%) of differentially expressed gene transcripts were down-regulated on average in the ovary of females producing poor egg quality spawns (**Table S2**).

**Figure 3:**
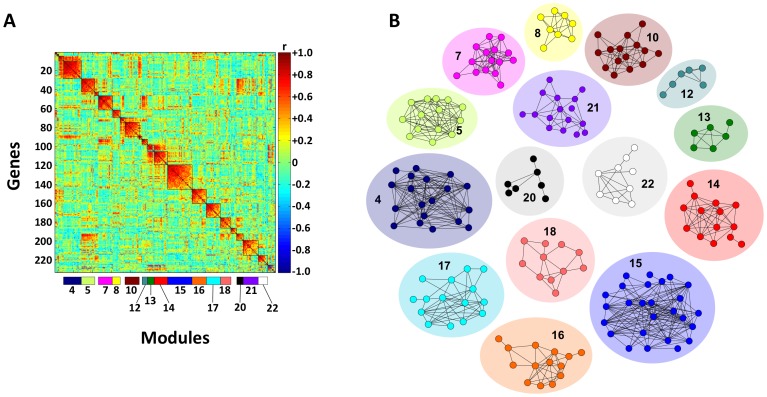
Modulated modularity clustering (MMC) heat map revealing modules of genes that covary in expression and relevance networks for genes within the modules. (A) MMC analysis of transcript expression levels for the top 233 genes identifying 23 gene modules. (B) Relevance networks for genes correlated within MMC modules obtained by enforcing an absolute correlation threshold of |r|≥0.6. Only networks containing 5 or more genes are depicted. Color-coding of the relevance networks by gene module corresponds to that shown in the horizontal bar under the heat map in (**A**).

When the 233-UniGene dataset was subjected to MMC, 23 modules of genes that inherently co-vary in expression after the effects of egg quality have been removed were evident (**Fig.3A**). When expression data for genes correlated within these transcriptional modules (|r|≥0.6) was fed into Cytoscape, several discrete gene relevance networks were revealed (**Fig 3B**) and their physiological significance was explored by detailed annotation of their composite genes. Among the 233 informative UniGenes, Blastx comparisons revealed 148 orthologues, of which 133 (57.3%) could be annotated with GO terms.

**Figure 4:**
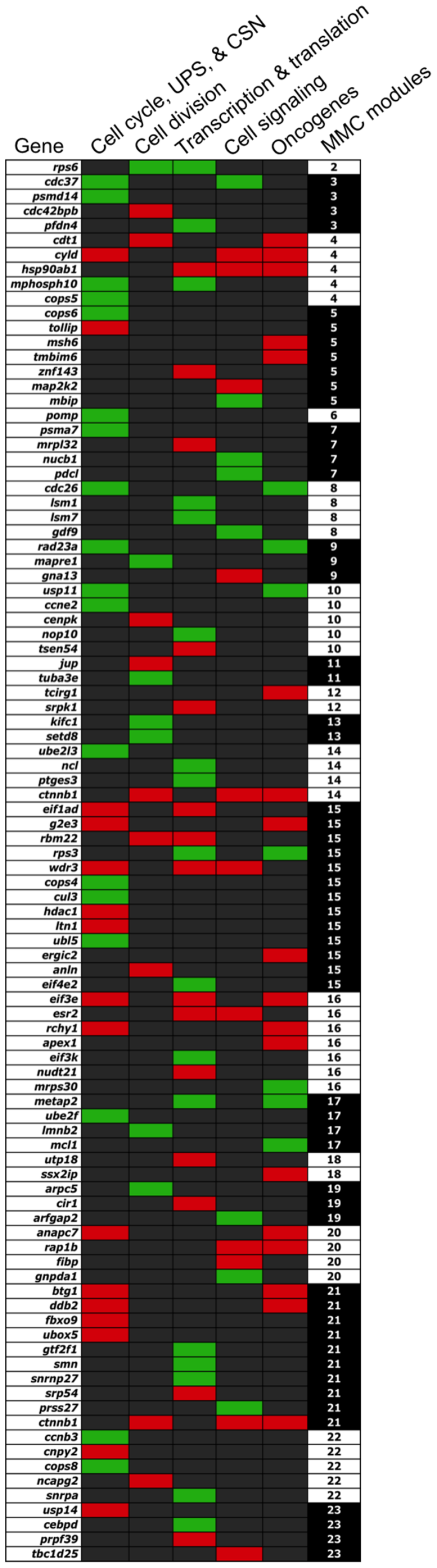
Matrix of a subset of genes that co-vary in expression and share GO classes or functional pathways. Presence of a red or green box indicates assignment of a gene to a functional category and up- or down-regulation, respectively, in fish spawning poor quality eggs (see also **Table S2**). MMC module indicates gene assignment to networks depicted in Fig. 3.

Of the 133 informative and annotated UniGenes, 34 (25.6%) were directly related to regulation of cell cycle (*ccnb3*, *hdac1*, *eif1ad*, *ccne2*, *mphosph10*, *cdc26*, *btg1*, *wdr3*, *anapc7*, *cdc37*, and *cyld*), or were genes that modulate the functions of such cell cycle regulators including subunits of the CSN (*cops4*, *cops5*, *cops6*, and *cops8*) and UPS, which includes the 26S-proteasome (*psmd14*, *pomp*, and *psma7*) and SKP1-cullin-F-box complex (SCF), cullin-RING ubiquitin ligases (CRL), and E2 ubiquitin-conjugating and E3 ubiquitin-ligase enzymes (*ubl5*, *rchy1*, *cnpy2*, *ube2l3*, *cul3*, *ltn1*, *g2e3*, *ddb2*, *fbxo9*, *ubox5*, *rad23a*, *usp11*, *cdc26*, *tollip*, *ube2f*, *anapc7*, *cyld*, *usp14*, and *eif3e*) (**Fig. 4**). Two of these transcripts (*g2e3* and *cyld*) along with three others identified in the dataset (*mcl1*, *tmbim6* and *mrps30*) encode apoptotic factors possibly related to the caspase 3 pathway of apoptosis induced by high cellular levels of ubiquinated-proteins that are not appropriately degraded by the UPS [43]. Remarkably, all of the 26S-proteasome and CSN components along with two cyclins (*ccnb3* and *ccne2*) were generally down-regulated in the ovary of females spawning poor quality eggs (**Table S2**).

Genes directly involved in the process of physical cell division represented 12.8% of the informative genes and included those involved in cytoskeletal formation of the mitotic spindle, DNA replication, chromosome condensation and migration, organelle remodeling and cytokinesis (*cdt1*, *rps6*, *setd8*, *ncapg2*, *cenpk*, *anln*, *mapre1*, *cdc42bpb*, *kifc1*, *rbm22*, *tuba3e*, *ctnnb1*, *lmnb2*, *arpc5*, *jup*, *hdac1*, *tlk1*, and *cdt1*) (**Fig. 4**).

Additional genes encode DNA repair proteins (*rps3*, *apex1*, *msh6*, *ddb2*, *rad23a*, and *usp11*). Twnty five (25) known oncogenes or genes whose expression is directly related to formation of malignant tumors also were identified (*anapc7*, *btg1, cdc26*, *cdt1*, *ctnnb1*, *cyld*, *ddb2*, *rchy1*, *metap2*, *eif3e*, *rap1b*, *rps3*, *ssx2ip*, *ergic2*, *mcl1*, *msh6*, *tcirg1*, *tmbim6*, *rad23a usp11*, *g2e3*, *apex1*, *metap2*, *mrps30*, and *hsp90ab1*), and approximately 72.0% of these were, on average, up-regulated in fish that spawned poor quality eggs (**Fig. 4** **, see also Table S2**).

Thirty-two (24.1%) of the informative genes encode products that either regulate transcription, translation, or hnRNA editing as part of the spliceosome, including *cebpd*, *cir1*, *eif1ad*, *eif3e*, *eif3k*, *eif4e2*, *esr2*, *gtf2f1*, *hsp90ab1*, *lsm1*, *lsm7*, *metap2*, *mphosph10*, *mrpl32*, *ncl*, *nop10*, *nudt21*, *pfdn4*, *prpf39*, *ptges3*, *rbm22*, *rps3*, *rps6*, *smn*, *snrnp27*, *snrpa*, *srp54*, *srpk1*, *tsen54*, *utp18*, *wdr3*, *znf143* (**Fig. 4**).

The last major class of genes encode growth factors or act in signal transduction or cell signaling (14.3% of the informative genes) and includes: *arfgap2*, *cdc37*, *ctnnb1*, *cyld*, *esr2*, *fibp*, *gdf9*, *gna13*, *gnpda1*, *hsp90ab1*, *map2k2*, *mbip*, *nucb1*, *pdcl*, *prss27*, *rap1b*, *tbc1d25*, and *wdr3* (**Fig. 4**).

## Discussion

In the present study, we examined the relationship between reproductive dysfunction and the ovary transcriptome in domesticated striped bass (*Morone saxatilis*), a species commonly exhibiting poor egg quality made manifest by early embryo developmental failure [24] (see **Fig. 1A**), which is a major problem in fish breeding programs. The female striped bass, which were matched for age, length and weight, were subjected to ovarian biopsy just before the breeding season and bred using standard hatchery procedures, and the quality of their spawns was judged by the percentage of eggs bearing viable embryos at 4 and 24 h following fertilization. All significant losses of embryos occurred in the first 4 h following fertilization, prior to MBT (**Fig.1A**), during which time ontogeny is dependent on maternal gene transcripts stored within the ovulated egg [44]. Remarkably, when mRNA extracted from the ovary biopsies was subjected to microarray and the data was analyzed using ANNs, models of the relation of ovary transcriptome to egg quality revealed that gene expression measured by as few as 250 probes, representing <2% of the ovary transcriptome queried, could explain most variation (>90%) in embryo survival (**Fig. 1B**). Furthermore, the ANN CV results showed that ∼80% of variation in 4 h embryo survival could be predicted from gene expression measured using just the top 250 probe dataset (mean R^2^ ∼0.80). Not surprisingly, using additional probes (1469) did not improve model predictions, possibly because little scope in unexplained variation in egg quality remained, but it has been our experience that overly complex models train the ANN to the idiosyncrasies of the input data and are not robust to CV. Using fewer probes (100) resulted in a decrease in the predictive ability (CV R^2^) of the models indicating that fertility depends upon the concerted action of many genes and it is quite possible to reduce the gene set so that model accuracy and robustness degrades.

Scrutiny of gene expression patterns evaluated using the 233 most informative UniGenes revealed a conspicuous and unusual transcript profile associated with egg quality that was clearly evident on the fine resolution heat map of gene expression (**Fig. 2**). In this profile, transcript abundance generally varies inversely between females producing high versus low quality spawns, although expression varies little (<0.2-fold) for individual genes and, in the ANN models, individual transcripts make minor contributions to overall prediction of egg quality (<1% of the variation). Such small collective changes in magnitude of gene expression resulting in striking predictive effects have been similarly been reported for honeybee behavior [45]. Interestingly, only one contig (#10015, *hsp90*) of the 66 (out of 11,208) contigs identified as having particularly abundant ovary expression in our prior report on the striped bass ovary transcriptome [36] was represented among the 233 transcripts whose collective levels predicted egg quality in the present study. This observation underscores the fact that highly expressed genes or genes that undergo large changes in expression are not necessarily the best predictors of complex phenotypic traits, such as egg quality.

The heat map also revealed some variation in expression of discrete genes between individual fish within egg quality groups, implying that certain regulatory pathways leading to transcriptome dysfunction may be activated to different extents among females or that differences in these pathways are not universally shared in all fish of either group. Nonetheless, as noted, the collective minor changes in expression of many score of genes (e.g. 233 UniGenes, 250 probes including duplicates) show a particularly powerful relationship to egg quality, measured as embryo developmental competence (**Fig. 1B**). Additionally, the majority of differentially expressed gene transcripts were down-regulated on average in the ovary of females producing poor egg quality spawns (62.2%), suggesting that the basis of this phenomenon may be insufficiency as opposed to overcompensation of maternal effects. In human and non-human primates, some oocytes with reduced developmental competence contain gene transcripts that fail to undergo proper post-transcriptional regulation during ovary maturation [46]. Our results indicate that developmental competence of eggs can be predicted from the stockpile of transcripts already present within the ovary before maturation, therefore such dysregulation may be the result of complex programming occurring throughout the different stages of oocyte growth.

Traditional linear-based analyses of microarray data aiming to identify a few candidate genes whose expression is related to egg quality may offer limited resolution. In the present study, the collective expression of at least 233 UniGenes was required to accurately predict egg quality, whereas using only 100 genes was less predictive (**Fig. 1B**). Furthermore, none of the differences in ovary gene expression identified by the ANN were found to be statistically significant by ANOVA (α = 0.05) once adjustments for multiple tests were considered. Sensitivity analyses, which extract the sensitivity of the output to changes in individual inputs [26], showed that no single gene accounted for more than 2% of the variation in egg quality explained via ANN modeling. These findings clearly indicate that the predictive power of the transcriptome is not due to individual genes, but rather to the information contained in their collective, coordinated behaviors, perhaps more appropriately evaluated as a transcriptomic “fingerprint”.

Genes can be assigned more than one Gene Ontology (GO) class, as the proteins they encode may perform multiple functions in more than one pathway [39]. However function or dysfunction of a particular gene in a physiological process influencing egg quality may be relegated to a specific subset of GOs. As multiple gene pathways may be functionally linked, a holistic analytical approach is required for proper biological interpretation. We used modulated modularity clustering (MMC) [27] to construct modules of genes that inherently co-vary in expression after the effects of egg quality have been removed, and gene relevance networks were generated from the resultant 23 transcriptional modules (**Fig. 3**). Based on gene annotations and MMC, we identified in the ovary of females producing poor egg quality spawns a dysfunction of the central collective apparatus of the ubiquitin-26S proteasome (UPS), COP9 signalosome (CSN), and cell cycle, suggesting that uncoordinated control of cell division is the primary cause of early embryo mortality prior to MBT.

Genes ultimately related to cell division (n = 96) accounted for 72.2% of the informative genes (n = 133) correlated to egg quality and more than half of these (54.2%) are represented in MMC modules 4, 5, 10, 15, 16, 21 and 22 (**Figs. 3** **and** **4**), indicating the importance of these seven transcriptional networks in early embryogenesis. This prevalence of genes involved in cell division that are present and co-expressed in discrete modules of the oocyte transcriptome appears to be highly conserved among vertebrates, as evidenced by the fact that the top representative GO terms for the three oocyte-specific gene modules identified by Weighted Gene Co-expression Network Analysis (rather than MMC) for both human and mouse preimplantation development were cell cycle, mitosis, and cytoskeleton [16]. Transcripts present in these modules, most of which were from genes involved in regulation of the cell cycle, transcription and RNA processing, were over represented in oocytes and progressively depleted during embryonic development, beginning as early as the 2-cell embryo stage. In the present study, most of the remaining informative genes (12.4%) encoded products involved in basal metabolic processes.

The role of the cell cycle as a master timer that coordinates the oocyte-to-zygote transition has been suggested [44] and our results confirm this, as the apparent dysregulation correlated with embryo mortality prior to MBT. The UPS intimately controls the cell cycle through precisely timed destruction of short-lived regulatory proteins including cyclins (ccns), cyclin-dependent kinases (cdks), and cell division cycle proteins (cdcs) during phase progression and at checkpoints of the cell cycle [47] (**Fig. S1**). This is elaborately mediated through mitogen-activated protein (MAP) kinase, stress-activated protein kinase/c-Jun NH_2_-terminal kinase (SAPK/JNK), CRL, SCF and the CSN signaling paths [48]–[50]. As the majority of these different regulatory components are spread across 7 MMC modules (**Fig. 4**), it is apparent that pathology of developmental incompetence is not only complex and integrated, but may manifest at different points during the cell cycle, presumably as the result of different causative factors. These components may in turn be regulated or their activities mediated by the transcription and translation factors that co-vary within these key MMC modules. For example, the CSN and 26S-proteasome both interact with eukaryotic translation initiation factors [51]–[52], four of which appear co-variable in MMC modules 15 and 16 (*eif1ad*, *eif3e*, *eif3k*, and *eif4e2*; **Fig. 4**). Additionally, transcription and translation factors also are generally believed to play important roles during the MZT [1].

The collective results of the present study supply powerful evidence that the ovary transcriptome may be a dominant factor contributing to egg quality. These findings also provide the first panoptic linkage of the various cell cycle regulatory components underpinning egg quality and, therefore, female reproductive fitness. Because the transcripts measured in ovary biopsy samples were taken prior to oocyte maturation and spawning, the observed transcriptomic differences were not a result of physical or physiological manifestations of embryo mortality but, rather, they are a feasible cause of these events. This mode of sampling offers the ability to select females *a priori* for breeding based on ovary gene expression profiles that are predictive of embryo developmental competency. Due to the evolutionary conservation of the gene pathways involved, their dysregulation may be an important molecular feature of reproductive failure in all vertebrates.

Although the proximal cause of transcriptome defects associated with developmental incompetence remains unclear, pedigree is unlikely to have a major influence as natural selection would act to eliminate subfertile individuals and our limited survey of microsatellite loci did not reveal any association of genotype with transcriptome or fertility. However, it is possible that a more exhaustive search of genetic polymorphisms (e.g. SNP's) might discover some loci linked to fertility [16]. We are presently replicating our experiments on additional groups of domesticated and wild striped bass employing direct sequencing (RNA-Seq), which will expand our coverage of the transcriptome while revealing a vast number of SNP's embedded in the transcripts to better examine potential impacts of allelic variation. The genes (n = 48) related to transcription, translation, cell signaling and signal transduction (**Fig. 4**) are potential candidates for identification of such proximal causes as their rates of expression may be influenced under specific environmental conditions. Associated epigenetic modifications of the DNA could underpin the correlation between transcript profiles and fertility, but examining this hypothesis will require more genomic information on striped bass than is currently available. Application of our ANN approach to discover the relation(s) between egg transcriptome and fertility in model species with more extensive genomic resources and knowledge of epigenetics, such as zebrafish [53], may be advantageous in this regard.

Finally, our results suggest that caution be used when employing spawning stock biomass (SSB) to indicate the reproductive health of commercially exploited fish stocks. The maturity schedule of females, upon which SSB computations are based, is usually estimated as the percentage of fish in each size or age class bearing growing or maturing oocytes or eggs, with the reproductive potential of such females being estimated based on their body mass. However, we observed large differences in egg quality and its unique transcriptomic fingerprint between striped bass matched for age, size (mass), and reproductive status. If similar differences exist within or between cohorts of wild fishes, SSB may over estimate reproductive potential because differences between females in egg quality are not taken into account. This takes on added significance if transcriptomic fingerprints shift toward higher fertility profiles with increasing age as, despite evidence to the contrary [54], most current fishery management models consider many small females to be equivalent to a few larger ones if the ovarian biomass is comparable. Transcriptomic profiling may provide an expedient means to better assess the reproductive health of fisheries and other animal resources important to global food security and has far reaching implications in agriculture and in medical fields such as human assisted reproductive technology.

## Conclusions

We discovered that it is possible to predict the state of a biologically complex fitness trait, egg quality, with remarkable accuracy based solely on ovary gene expression profiles. The collective expression of a discrete suite of ovarian transcripts, constituting a transcriptomic fingerprint, involved specific, highly-conserved gene networks central to early embryonic development in all vertebrates. Our findings open the door to ‘transcriptome-assisted’ breeding and assessment of reproductive condition and value in farmed and possibly wild fishes and other vertebrates, possibly including human assisted reproductive technologies. The ovary transcriptome profiles and ANN analytical methods developed in the present study provide a foundation for future research into the effects of perturbations (e.g., selection pressure, environment, husbandry practice) on egg transcriptome and quality, so that gene networks and molecular pathways most susceptible to a particular insult can be identified and corrective or mitigating measures implemented. Additionally, we propose that ANNs may be a superior alternative to linear-based analyses of gene expression data that can be used as a diagnostic tool, provided that the ANNs are trained with appropriate data.

## Supporting Information

Figure S1 **Model depicting the interactions of important pathways that ultimately influence cell division and that are implicated in egg quality of striped bass.**
(TIF)

Table S1 Microsatellite loci used for genotyping and National Center for Biotechnology Information (NCBI) accession information.
(DOCX)

Table S2 Differential of expression of genes (n = 233) in the ovaries of females producing low versus high egg quality spawns.
(DOCX)
