# Supplementary figures and images for: Ovary Transcriptome Profiling via Artificial Intelligence Reveals a Transcriptomic Fingerprint Predicting Egg Quality in Striped Bass, Morone saxatilis
Source: PLoS One. 2014 May 12;9(5):e96818. doi: 10.1371/journal.pone.0096818 (PMC4018430; doi:10.1371/journal.pone.0096818)

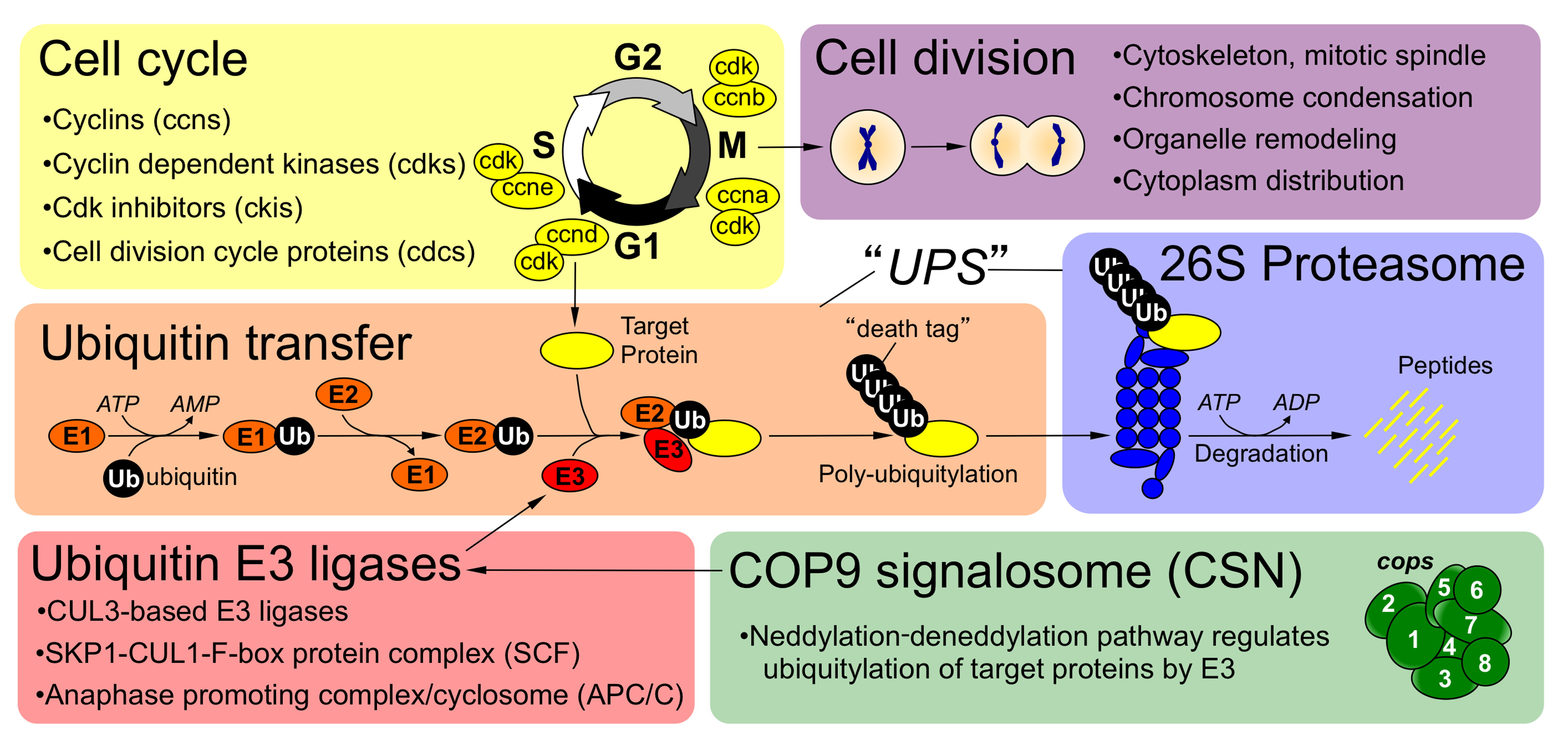

Supplement: Figure S1 — Model depicting the interactions of important pathways that ultimately influence cell division and that are implicated in egg quality of striped bass. (TIF) [file pone.0096818.s001.tif]
