# Supplementary material for: Ovary Transcriptome Profiling via Artificial Intelligence Reveals a Transcriptomic Fingerprint Predicting Egg Quality in Striped Bass, Morone saxatilis
Source: PLoS One. 2014 May 12;9(5):e96818. doi: 10.1371/journal.pone.0096818 (PMC4018430; doi:10.1371/journal.pone.0096818)
Supplement: Table S1 — Microsatellite loci used for genotyping and National Center for Biotechnology Information (NCBI) accession information. (DOCX) [file pone.0096818.s002.docx]

**Table S1**. Microsatellite loci used for genotyping and National Center for Biotechnology Information (NCBI) accession information.

| **Microsatellite**  **Locus** | **NCBI**  **UniSTS** | **GenBank**  **Accession** |
| --- | --- | --- |
| MSM1084 | 477540 | BV678170 |
| MSM1087 | 477543 | BV678173 |
| MSM1093 | 477546 | BV678176 |
| MSM1110 | 477561 | BV678191 |
| MSM1123 | 477571 | BV678200 |
| MSM1132 | 477576 | BV678205 |
| MSM1134 | 477578 | BV678206 |
| MSM1138 | 477582 | BV678652 |
| MSM1142 | 477585 | BV678212 |
| MSM1148 | 477591 | BV678218 |
| MSM1159 | 477602 | BV678656 |
| MSM1161 | 477603 | BV678228 |
| MSM1186 | 477626 | BV678307 |
| MSM1192 | 477629 | BV678263 |
| MSM1196 | 477633 | BV678267 |
| MSM1213 | 477645 | BV678250 |
| MSM1224 | 477655 | BV678260 |
| MSM1228 | 477657 | BV678271 |
| MSM1236 | 477665 | BV678659 |
| MSM1241 | 477670 | BV678279 |
| MSM1246 | 477675 | BV678290 |
| MSM1439 | 477840 | BV678471 |
| MSM1442 | 477843 | BV678474 |
| MSM1458 | 477858 | BV678489 |
| MSM1471 | 477870 | BV678501 |
| MSM1481 | 477878 | BV678509 |
| MSM1493 | 477890 | BV678521 |
| MSM1516 | 477890 | BV678521 |
| MSM1519 | 477915 | BV678546 |
| MSM1530 | 477925 | BV678556 |
| MSM1534 | 477928 | BV678559 |
| MSM1562 | 477952 | BV678582 |
| MSM1566 | 477956 | BV678586 |
| MSM1575 | 477964 | BV678594 |
| MSM1586 | 477973 | BV678603 |
| MSM1588 | 477975 | BV678605 |
