# Supplementary material for: Ovary Transcriptome Profiling via Artificial Intelligence Reveals a Transcriptomic Fingerprint Predicting Egg Quality in Striped Bass, Morone saxatilis
Source: PLoS One. 2014 May 12;9(5):e96818. doi: 10.1371/journal.pone.0096818 (PMC4018430; doi:10.1371/journal.pone.0096818)
Supplement: Table S2 — Differential of expression of genes (n = 233) in the ovaries of females producing low versus high egg quality spawns. (DOCX) [file pone.0096818.s003.docx]

**Table S2**. Differential expression^1^ of genes (n=233) in the ovaries of females producing low versus high egg quality spawns.

| **Probe Name** | **MMC** | **Diff LvsH** | ***HUGO*** |
| --- | --- | --- | --- |
| contig09968\|mitochondrial | 7 | 0.18976 | *mrpl32* |
| contig07452\|---NA--- | 19 | 0.17367 | novel |
| contig07636\|---NA--- | 14 | 0.17339 | novel |
| contig00925\|short-chain | 17 | 0.17265 | *acads* |
| contig04033\|f-box | 21 | 0.13965 | *fbxo9* |
| contig02985\|testis | 5 | 0.13695 | *tmbim6* |
| contig10206\|---NA--- | 17 | 0.12847 | novel |
| contig06504\|tripartite | 6 | 0.12751 | *trim2* |
| contig04487\|actin | 15 | 0.12748 | *anln* |
| contig06568\|trna | 10 | 0.1173 | *tsen54* |
| contig04811\|guanine | 9 | 0.11217 | *gna13* |
| contig03704\|damage-specific | 21 | 0.11115 | *ddb2* |
| contig01331\|---NA--- | 6 | 0.11024 | novel |
| contig08442\|cbf1 | 19 | 0.10774 | *cir1* |
| contig03458\|sfrs | 12 | 0.10689 | *srpk1* |
| contig06481\|aspartyl-trna | 17 | 0.10472 | *dars* |
| contig01020\|---NA--- | 7 | 0.10467 | novel |
| contig09509\|rna | 15 | 0.10271 | *rbm22* |
| contig00539\|zinc | 5 | 0.10211 | *znf143* |
| contig02141\|g2 | 15 | 0.10193 | *g2e3* |
| contig00710\|thimet | 19 | 0.10171 | *thop1* |
| contig02135\|gamma-glutamyl | 20 | 0.10035 | *ggcx* |
| contig10015\|heat | 4 | 0.097643 | *hsp90ab1* |
| contig01439\|ergic | 15 | 0.09663 | *ergic2* |
| contig04603\|---NA--- | 4 | 0.094522 | novel |
| contig00501\|small | 18 | 0.094202 | *utp18* |
| contig10034\|---NA--- | 18 | 0.092736 | novel |
| contig01400\|solute | 17 | 0.092509 | *slc25a12* |
| contig01757\|junction | 11 | 0.091912 | *jup* |
| contig02916\|cleavage | 16 | 0.090671 | *nudt21* |
| contig08169\|prpf39 | 23 | 0.089997 | *prpf39* |
| contig02207\|eukaryotic | 15 | 0.08946 | *eif1ad* |
| contig05245\|dpcd | 12 | 0.089135 | *dpcd* |
| contig07705\|high | 21 | 0.086861 | *hdlbp* |
| contig09260\|cylindromatosis | 4 | 0.085891 | *cyld* |
| contig08283\|---NA--- | 5 | 0.085634 | novel |
| contig01161\|endonuclease | 16 | 0.085413 | *apex1* |
| contig03927\|tetratricopeptide | 7 | 0.084932 | *ttc27* |
| contig04693\|solute | 17 | 0.083333 | *slc38a8* |
| contig03766\|cdt1 | 4 | 0.081967 | *cdt1* |
| contig03108\|chromosome | 5 | 0.080478 | *c19orf10* |
| contig07542\|anapc7 | 20 | 0.080413 | *anapc7* |
| contig08151\|transmembrane | 17 | 0.079889 | *tmbim4* |
| contig01001\|sphingosine-1-phosp... | 14 | 0.077658 | *sgpp1* |
| contig10439\|ubiquitin | 23 | 0.076887 | *usp14* |
| sequence04\|MoroneRestrogenRestr… | 16 | 0.076732 | *esr2* |
| contig03116\|cenpk | 10 | 0.076119 | *cenpk* |
| contig02106\|phosphoserine | 15 | 0.075762 | *psph* |
| contig06020\|---NA--- | 16 | 0.074964 | novel |
| contig07923\|---NA--- | 5 | 0.07295 | novel |
| contig01993\|zinc | 15 | 0.071472 | *ltn1* |
| contig11154\|non-structural | 21 | 0.071099 | *nsmce1* |
| contig06247\|toll | 5 | 0.070389 | *tollip* |
| contig01291\|canopy | 22 | 0.06892 | *cnpy2* |
| contig07451\|wd | 15 | 0.068767 | *wdr3* |
| contig02580\|nitric | 10 | 0.067996 | *nosip* |
| contig05068\|dead | 7 | 0.067671 | *ddx52* |
| contig07736\|cdc42 | 3 | 0.067593 | *cdc42bpb* |
| contig02527\|signal | 21 | 0.067135 | *srp54* |
| contig03182\|---NA--- | 15 | 0.065596 | novel |
| contig09163\|---NA--- | 14 | 0.065531 | novel |
| contig00718\|fibroblast | 20 | 0.064772 | *fibp* |
| contig03670\|t-cell | 12 | 0.063314 | *tcirg1* |
| contig01254\|histone | 15 | 0.062888 | *hdac1* |
| contig06348\|b-cell | 21 | 0.061853 | *btg1* |
| contig00271\|ring | 16 | 0.061786 | *rchy1* |
| contig00343\|eukaryotic | 16 | 0.060476 | *eif3e* |
| contig06418\|mitogen-activated | 5 | 0.060265 | *map2k2* |
| contig01503\|leucine | 22 | 0.059402 | *ncapg2* |
| contig04602\|tbc1 | 23 | 0.05914 | *tbc1d25* |
| contig02942\|golgi | 4 | 0.05897 | *gorasp2* |
| contig05887\|member | 20 | 0.058683 | *rap1b* |
| contig04441\|u-box | 21 | 0.058304 | *ubox5* |
| contig02843\|muts | 5 | 0.056302 | *msh6* |
| contig01348\|synovial | 18 | 0.055663 | *ssx2ip* |
| contig04500\|unnamed | 4 | 0.054656 | *** |
| contig05425\|---NA--- | 15 | 0.053111 | novel |
| contig09064\|catenin | 21 | 0.0531 | *ctnnb1* |
| contig08561\|---NA--- | 14 | 0.052798 | novel |
| contig09064\|catenin | 14 | 0.051163 | *ctnnb1* |
| contig08548\|---NA--- | 13 | 0.048949 | novel |
| contig00593\|gpn-loop | 4 | 0.048001 | *gpn2* |
| contig05208\|unnamed | 18 | 0.046966 | *** |
| contig06058\|foie | 3 | 0.046812 | *c4orf41* |
| contig08981\|carnitine | 16 | 0.046545 | *crot* |
| contig09476\|---NA--- | 15 | 0.04654 | novel |
| contig00452\|transmembrane | 10 | 0.043443 | *tmco7* |
| contig01368\|aldehyde | 7 | 0.036055 | *aldh3b1* |
| contig05847\|uv | 9 | -0.020602 | *rad23a* |
| contig01428\|prefoldin | 3 | -0.024811 | *pfdn4* |
| contig09690\|---NA--- | 15 | -0.025649 | novel |
| contig02089\|survival | 21 | -0.031005 | *smn1* |
| contig03079\|adaptor-related | 22 | -0.031833 | *ap2s1* |
| contig03876\|---NA--- | 4 | -0.038664 | novel |
| PG61-2\|MoroneR---NA---R---NA--- | 15 | -0.039512 | novel |
| contig00701\|melanoma | 14 | -0.039626 | *mum1l1* |
| VG59-11\|Morone\|---NA---\|---NA--- | 2 | -0.040242 | novel |
| contig00793\|---NA--- | 17 | -0.042697 | novel |
| contig05619\|---NA--- | 10 | -0.042916 | novel |
| contig00518\|f11 | 16 | -0.044037 | *f11r* |
| contig00067\|lsm7 | 8 | -0.045531 | *lsm7* |
| contig00081\|mitochondrial | 16 | -0.045868 | *mrps30* |
| contig11099\|glutathione | 17 | -0.045876 | *gpx1* |
| contig00615\|fam46c | 18 | -0.047661 | *fam46c* |
| contig02643\|serine | 21 | -0.047859 | *prss27* |
| contig03637\|---NA--- | 14 | -0.048174 | novel |
| contig06420\|ubiquitin-conjugating | 17 | -0.048783 | *ube2f* |
| contig06312\|---NA--- | 10 | -0.049536 | novel |
| contig02133\|---NA--- | 21 | -0.051397 | novel |
| contig00903\|hematopoietic | 1 | -0.052828 | *c19orf63* |
| contig02428\|cop9 | 5 | -0.054781 | *cops6* |
| contig00831\|---NA--- | 9 | -0.054825 | novel |
| contig03288\|---NA--- | 16 | -0.054839 | novel |
| contig08472\|cell | 3 | -0.05574 | *cdc37* |
| contig00511\|proteasome | 3 | -0.055931 | *psmd14* |
| contig02287\|---NA--- | 11 | -0.056432 | novel |
| contig05769\|---NA--- | 10 | -0.056564 | novel |
| contig05564\|---NA--- | 4 | -0.056654 | novel |
| contig02656\|myeloid | 17 | -0.057183 | *mcl1* |
| contig06305\|---NA--- | 14 | -0.05872 | novel |
| contig02990\|---NA--- | 17 | -0.058929 | novel |
| contig09270\|phosducin-like | 7 | -0.060048 | *pdcl* |
| contig02573\|proteasome | 7 | -0.060279 | *psma7* |
| contig01146\|cop9 | 15 | -0.060373 | *cops4* |
| contig00591\|nucleolin | 14 | -0.06083 | *ncl* |
| contig02725\|---NA--- | 9 | -0.06141 | novel |
| contig06189\|---NA--- | 4 | -0.061843 | novel |
| contig00301\|methionyl | 17 | -0.062352 | *metap2* |
| contig00070\|ubiquitin-like | 15 | -0.062368 | *ubl5* |
| contig03957\|cop9 | 22 | -0.062456 | *cops8* |
| VG6a-1\|Morone\|glucosamine-6-pho... | 20 | -0.062458 | *gnpda1* |
| contig00137\|n-terminal | 10 | -0.062478 | *naa10* |
| contig02312\|---NA--- | 4 | -0.06473 | novel |
| contig07631\|---NA--- | 14 | -0.064958 | novel |
| contig06729\|---NA--- | 13 | -0.065008 | novel |
| contig01335\|predicted | 16 | -0.065535 | *** |
| contig01855\|proteasome | 6 | -0.066469 | *pomp* |
| contig09401\|---NA--- | 10 | -0.06729 | novel |
| contig00576\|---NA--- | 20 | -0.067471 | novel |
| contig08085\|---NA--- | 15 | -0.068124 | novel |
| contig04999\|---NA--- | 7 | -0.068138 | novel |
| contig03237\|nadh | 5 | -0.069779 | *ndufa7* |
| contig05387\|---NA--- | 5 | -0.069912 | novel |
| contig10402\|cytochrome | 21 | -0.070667 | *cyc1* |
| contig05914\|---NA--- | 4 | -0.070806 | novel |
| contig05209\|eukaryotic | 15 | -0.071154 | *eif4e2* |
| contig03619\|---NA--- | 5 | -0.071187 | novel |
| contig02488\|---NA--- | 8 | -0.071722 | novel |
| contig09024\|kinesin | 13 | -0.071908 | *kifc1* |
| contig01059\|---NA--- | 18 | -0.07254 | novel |
| contig00530\|---NA--- | 15 | -0.072666 | novel |
| contig00614\|cyclin | 22 | -0.072915 | *ccnb3* |
| contig05304\|map3k12 | 5 | -0.073197 | *mbip* |
| contig09372\|---NA--- | 7 | -0.073478 | novel |
| contig00574\|eh | 16 | -0.073812 | *ehd3* |
| contig02086\|---NA--- | 15 | -0.073944 | novel |
| contig09521\|---NA--- | 20 | -0.074059 | novel |
| contig07554\|---NA--- | 12 | -0.074537 | novel |
| contig09629\|---NA--- | 22 | -0.074917 | novel |
| contig09444\|succinate | 8 | -0.074992 | *sdha* |
| contig01379\|ubiquitin-conjugating | 14 | -0.075048 | *ube2l3* |
| contig02764\|---NA--- | 7 | -0.075248 | novel |
| contig01412\|cop9 | 4 | -0.07529 | *cops5* |
| contig07418\|---NA--- | 4 | -0.075575 | novel |
| contig07059\|---NA--- | 6 | -0.075954 | novel |
| contig08047\|trafficking | 16 | -0.07628 | *trappc3* |
| contig00319\|set | 13 | -0.076916 | *setd8* |
| contig09958\|growth | 8 | -0.077431 | *gdf9* |
| contig01657\|nucleolar | 21 | -0.07838 | *snrnp27* |
| contig04124\|general | 21 | -0.078894 | *gtf2f1* |
| contig04655\|nadh | 12 | -0.079261 | *ndufs5* |
| contig05421\|---NA--- | 18 | -0.079762 | novel |
| contig05099\|m-phase | 4 | -0.080044 | *mphosph10* |
| contig01640\|molybdenum | 7 | -0.080289 | *mocs2* |
| contig07041\|---NA--- | 15 | -0.080349 | novel |
| contig00729\|eukaryotic | 16 | -0.080457 | *eif3k* |
| contig00106\|---NA--- | 5 | -0.080619 | novel |
| contig04239\|dynactin | 13 | -0.080915 | *dctn6* |
| contig06373\|---NA--- | 18 | -0.081069 | novel |
| contig01495\|cullin | 15 | -0.081349 | *cul3* |
| contig05424\|rwd | 23 | -0.081644 | *rwdd2b* |
| contig00156\|cytochrome | 23 | -0.082073 | *uqcr11* |
| contig09237\|nucleobindin | 7 | -0.082158 | *nucb1* |
| contig02902\|---NA--- | 15 | -0.082501 | novel |
| contig02775\|tousled-like | 7 | -0.082971 | *tlk1* |
| contig04214\|ccaat | 23 | -0.083015 | *cebpd* |
| contig10667\|---NA--- | 4 | -0.083424 | novel |
| contig01089\|---NA--- | 1 | -0.085502 | novel |
| contig03514\|---NA--- | 10 | -0.086099 | novel |
| contig11148\|tubulin | 11 | -0.086424 | *tuba3e* |
| contig04133\|---NA--- | 4 | -0.086612 | novel |
| contig00655\|lsm1 | 8 | -0.086774 | *lsm1* |
| contig04816\|cyclin | 10 | -0.087225 | *ccne2* |
| contig07088\|---NA--- | 22 | -0.089133 | novel |
| contig07503\|---NA--- | 15 | -0.089202 | novel |
| contig03917\|---NA--- | 17 | -0.090485 | novel |
| contig09057\|trafficking | 8 | -0.091358 | *trappc4* |
| contig03338\|lipase | 15 | -0.09222 | *lmf2* |
| contig02512\|chromosome | 14 | -0.092452 | *** |
| contig01101\|ribosomal | 15 | -0.09253 | *rps3* |
| contig00239\|small | 22 | -0.093844 | *snrpa* |
| contig05305\|---NA--- | 4 | -0.094777 | novel |
| contig04373\|---NA--- | 7 | -0.095456 | novel |
| contig11165\|h | 10 | -0.096583 | *nop10* |
| contig08611\|---NA--- | 10 | -0.098682 | novel |
| contig05660\|---NA--- | 10 | -0.10164 | novel |
| contig05333\|---NA--- | 4 | -0.10343 | novel |
| contig05670\|---NA--- | 21 | -0.10375 | novel |
| contig02344\|prostaglandin | 14 | -0.10428 | *ptges3* |
| contig05516\|chromosome | 21 | -0.10551 | *c7orf59* |
| contig06508\|microtubule-associated | 9 | -0.10608 | *mapre1* |
| contig00142\|ribosomal | 2 | -0.10724 | *rps6* |
| contig10453\|---NA--- | 13 | -0.10837 | novel |
| contig03521\|arsa | 17 | -0.10857 | *asna1* |
| contig06838\|lamin | 17 | -0.10861 | *lmnb2* |
| contig06808\|wd | 12 | -0.10911 | *wipi2* |
| contig09598\|---NA--- | 18 | -0.11698 | novel |
| contig03920\|---NA--- | 14 | -0.12093 | novel |
| contig06742\|---NA--- | 23 | -0.12238 | novel |
| contig06242\|cell | 8 | -0.12349 | *cdc26* |
| contig00351\|cu051 | 7 | -0.12437 | *** |
| contig05513\|---NA--- | 23 | -0.12472 | novel |
| contig04722\|actin | 19 | -0.12956 | *arpc5* |
| contig07428\|adp-ribosylation | 19 | -0.13167 | *arfgap2* |
| contig03283\|dna | 18 | -0.13192 | *helq* |
| contig00954\|---NA--- | 22 | -0.1392 | novel |
| contig05608\|---NA--- | 8 | -0.13943 | novel |
| contig06215\|ubiquitin | 10 | -0.14091 | *usp11* |
| contig02027\|---NA--- | 18 | -0.14226 | novel |
| contig04912\|---NA--- | 13 | -0.14258 | novel |
| contig01584\|ubiquinol-cytochrome | 16 | -0.14923 | *uqcrh* |
| contig08570\|---NA--- | 19 | -0.19182 | novel |
| contig05668\|---NA--- | 22 | -0.1967 | novel |
| ^1^Genes are identified by microarray Probe Name. MMC indicates assigned module number as depicted in Fig. 4A & B. Diff LvsH indicates the average of the log(2) gene expression values generated by VSN for females producing low quality eggs minus this average for females producing high quality eggs, i.e. Diff LvsH indicates the log(2)-fold difference in gene expression between the two groups of fish. HUGO indicates the approved Human Genome Organization Gene Nomenclature Committee name for each gene. Genes are organized by magnitude of difference in expression “Diff LvsH” with values for genes upregulated in low egg quality spawners being positive and values for genes downregulated in low egg quality spawners being negative.  *novel = no BLASTx return (E-value < 10^-3)* | | | |
| ** = no annotation or approved HUGO gene name* | | | |
| Duplicate probes corresponding to catenin (*ctnnb1*) are shown in grey shaded boxes. | | | |
